# Supplementary material for: Infectiousness of Leishmania major to Phlebotomus papatasi: differences between natural reservoir host Meriones shawi and laboratory model BALB/c mice
Source: PLoS Negl Trop Dis. 2025 Jun 16;19(6):e0013183. doi: 10.1371/journal.pntd.0013183 (PMC12201674; doi:10.1371/journal.pntd.0013183)
Supplement: S1 Appendix — (DOCX) [file pntd.0013183.s001.docx]

**S1 Appendix. DNA mass from the blood meals of *Phlebotomus papatasi* and from skin microbiopsies.**

**METHODS**: Female *P. papatasi* were dissected within 1-3 hours post feeding on BALB/c mouse. Peritrophic matrix filled with blood meal were separated from the midgut epithelium and transferred into Eppendorf tubes containing 100 µl of tissue lysis buffer. Skin microbiopsies were taken by Harper devices from the same BALB/c mouse, as described in Materials and Methods chapter, and transferred into 100 µl of tissue lysis buffer. Samples were then isolated using the High Pure PCR Template Isolation Kit (Roche) according to the manufacturer’s instructions. DNA mass was measured by NanoDrop.

**RESULTS:**

| **DNA from *P. papatasi* blood meals (ng)** | **DNA from microbiopsies (ng)** |
| --- | --- |
| 45 | 35 |
| 80 | 40 |
| 65 | 42.5 |
| 55 | 45 |
| 80 | 62.5 |
| 55 | **AVERAGE: 45.0** |
| 42.5 |  |
| 62 |  |
| 57 |  |
| 82.5 |  |
| 162.5 (5 guts pool) |  |
| **AVERAGE: 52.4** |  |
