# Supplementary material for: Infectiousness of Leishmania major to Phlebotomus papatasi: differences between natural reservoir host Meriones shawi and laboratory model BALB/c mice
Source: PLoS Negl Trop Dis. 2025 Jun 16;19(6):e0013183. doi: 10.1371/journal.pntd.0013183 (PMC12201674; doi:10.1371/journal.pntd.0013183)
Supplement: Attachment — Submitted filename: Review PLOS Lm in Pp.pdf [file pntd.0013183.s005.pdf]

Review

Infectiousness of Leishmania major to Phlebotomus papatasi: differences between natural reservoir host Meriones shawi and laboratory model BALB/c mice

In this study, the authors show an interesting difference in the distribution of *Leishmania major* amastigotes in the skin of *Meriones shawi* and BALB/c mice revealed differences in infectiousness to sand flies. These findings underscore the importance of natural host models for a better understanding of CL transmission and more accurate epidemiological modeling. So, the current study is on a topic of relevance and general interest to the readers of the journal presenting originality and importance of the contribution for the development of the field of study.

The authors of this article have extensive experience in *Leishmania*-sand fly interactions and have provided valuable insights into the transmission dynamics of *Leishmania major*, particularly the role of *Meriones shawi* as a reservoir host.

The following are the reviews and questions that must be presented by the authors before publication:

Minor revisions

1- Introduction:

Line 16- The sentence should be revised: These reservoir animals are the main source of infection for vectors, phlebotomine sand flies (Diptera: Psychodidae), which act as vectors, transmitting parasites to humans, who are incidental hosts.

Please expand the introduction. Specifically, include information on how *P. papatasi* serves as a specific vector for *L. major*, highlighting key aspects of factors influencing vector competence.

2- Methods and Results

Experimental infections of rodents and Infectiousness of rodents at the microscale:

The experiment was terminated at 15–38 p.i. in Meriones shawi -

It is unclear what infection time points were analyzed for *M. shawi*, and the reason for the long time gap between the selected post-infection intervals is not explained. Could you clarify the rationale behind these choices?

The number of infected and asymptomatic animals analyzed appears to be low, with only three asymptomatic ears examined. Additionally, how does this sample size

impact the robustness of the conclusions regarding parasite load dynamics in *M. shawi*?

Fig 1 – The arrow in Figure 1 is not appearing. Please check and adjust the figure to ensure the arrow is clearly visible.

In Figure 3, panel (E) appears to express the data as percentages, whereas panel (F) seems to use relative numbers. To ensure consistency and clarity, you should express all data in percentages.

Specifically, 50.0% of the females that fed at the lesion centers and 58.3% of those that fed at the lesion margins acquired the infection (Fig 3F). The 50% infection rate is based on only four animals. Could the small number of insects used in this analysis influence the reliability of the results?

Fig 4. Schematic of the trend of parasite loads in MBs collected from M. shawi pinnae during the experiment. It would be more valuable to present a table with the MB values found rather than a schematic graph.

Fig 5. Infection rates of P. papatasi females fed different infective doses. Please add the missing 10% value on the Y-axis

I think that a histological or microscopic examination of the three zones of ulcerative lesions in both BALB/c mice and M. shawi at the same time points could provide valuable insights into the spatial dynamics of parasite distribution and tissue response.
