# Supplementary material for: Infectiousness of Leishmania major to Phlebotomus papatasi: differences between natural reservoir host Meriones shawi and laboratory model BALB/c mice
Source: PLoS Negl Trop Dis. 2025 Jun 16;19(6):e0013183. doi: 10.1371/journal.pntd.0013183 (PMC12201674; doi:10.1371/journal.pntd.0013183)
Supplement: Attachment — Submitted filename: Review report PLOS NTD 2025.docx [file pntd.0013183.s004.docx]

Re: Infectiousness of *Leishmania major* to *Phlebotomus papatasi*: differences between natural reservoir host *Meriones shawi* and laboratory model BALB/c mice

PNTD-D-25-00050

**Authors**: Vojtková et al.

**General Comments**

The authors highlighted the differences in infectiousness to sandflies between natural reservoir hosts and animal models. They found that using animal models to estimate the infectiousness of parasites to vectors tends to underestimate parasite transmission in leishmaniasis. They reported that only a few parasites from natural animal models could establish an infection in sandflies, in contrast to the much higher numbers observed in animal models needed to establish infection. These findings could enhance our understanding of *Leishmania* transmission.

While *Meriones shawi* is the natural reservoir host for *Leishmania* parasites, these animals remain in a laboratory setting for extended periods without exposure to sand flies and parasites. How do the authors relate the findings from this animal study to those in the wild, which experience higher exposure to sand-fly bites and parasite infections, concerning the parameters measured in the experiment? The immune response of hosts with frequent exposure might differ from those with no or limited exposure.

The study's objectives should be presented clearly and concisely.

The authors employed various statistical models to interpret the results; however, they did not specify the number of sandflies used in the infection experiments or the number of natural hosts and laboratory animals included in the study following the sample size calculation procedure. The first paragraph of the results section confuses methods and results.

Figure 4 is confusing because the authors have used different notations, such as 2x and 1x, without clear explanation.
