# Supplementary material for: Infectiousness of Leishmania major to Phlebotomus papatasi: differences between natural reservoir host Meriones shawi and laboratory model BALB/c mice
Source: PLoS Negl Trop Dis. 2025 Jun 16;19(6):e0013183. doi: 10.1371/journal.pntd.0013183 (PMC12201674; doi:10.1371/journal.pntd.0013183)
Supplement: Attachment — Submitted filename: Response to Reviewers.docx [file pntd.0013183.s008.docx]

We thank all the Reviewers for the favorable reviews, for the valuable suggestions and for the time spent evaluating the manuscript.

Reviewer #1: The manuscript PNTD-D-25-00050 entitled "Infectiousness of Leishmania major to Phlebotomus papatasi: differences between natural reservoir host Meriones shawi and laboratory model BALB/c mice." is a well designed study on the transmission parameters of L. major in two rodent models. Authors provide important aspects of the infection on the natural reservoir host of this parasite, comparing the obtained data with laboratory model rodents. The manuscript is well written, and deserves publication in PNTD pending minor revisions.
Bellow I provided some comments and suggestions to improve the quality of the manuscript.
Abstract:
-Use "ear pinnae" intead of "pinnae".

Corrected also in other parts of the manuscript
Introduction:
-Second paragraph: Please add some information on the vector of L. major. Also, replace the sentence "These reservoir animals are the main source of infection for vectors, phlebotomine sand flies (Diptera: Psychodidae), which act as vectors, transmitting parasites to humans, who are incidental hosts" by "These reservoir animals are the main source of infection for phlebotomine sand flies (Diptera: Psychodidae), which act as vectors, transmitting parasites to other vertebrate hosts, including humans, who are incidental hosts".

The sentence was corrected and following text was added to the second paragraph of Introduction: *Phlebotomus papatasi* is the principal vector of *L. major* over a wide geographical range that extends from North Africa to India, with breeding sites in rodent burrows (Maroli 2012). In this specific vector, parasite attachment to the midgut epithelium is controlled by the surface glycoconjugate lipophosphoglycan, which selectively binds to the midgut galectin receptor (Kamhawi 2006).

-Second paragraph: Add "For example," before "Heterogeneity".

Done.

-Last paragraph: The sentences "Amastigote numbers at bite sites were estimated via microbiopsies (MBs), which sampled a volume comparable to what the feeding female sand fly takes by her proboscis. The results from the natural host model were compared with those from a standard laboratory model using BALB/c mice." are methods and should be deleted from the introduction.

Done.

Methods:
-Please use the term "euthanasia" instead of "sacrifice".

Corrected. We originally used this term because in our national terminology the term euthanasia is reserved for human medicine.

Reviewer #2: The study's objectives should be presented clearly and concisely.
The authors employed various statistical models to interpret the results; however, they did not specify the number of sandflies used in the infection experiments or the number of natural hosts and laboratory animals included in the study following the sample size calculation procedure.

The specification of the number of sand flies was added to the first paragraph of the Results section “Infectiousness at the microscale”: “A total of 161 sand flies and MBs from bite sites in 24 *M. shawi* and 5 BALB/c mice were analyzed (S2 Table).”

Reviewer #3: Based on the overall quality of the study, the relevance of the topic, and the robustness of the data presented, the manuscript is suitable for publication pending minor revisions. The suggested clarifications and additional analyses will further strengthen the manuscript, enhancing the understanding of parasite distribution and infectiousness in natural and experimental models.

**Results**

-Reviewer #1: Results:
-This section could be better summarized as there are a lot of information that fits on material and methods. Please try to keep only the analytical part on the results section.

- Figure 3A and 3B are part of methodology and should be moved to the M&M section along with the text to which it was referred.

Two paragraphs of the Results section were reduced and the information moved to the Methods section, as have Figures 3A and 3B, which are now labeled Figure 1.

Reviewer #2: The first paragraph of the results section confuses methods and results.

The first paragraph has been moved to the Methods section.

Figure 4 is confusing because the authors have used different notations, such as 2x and 1x, without clear explanation.

Figure 4 has been removed and replaced with a table; we believe that this make the data easier to understand.

Reviewer #3: The results are generally well presented, but some methodological details, such as the rationale for infection time points and the low sample size of asymptomatic animals, require further explanation.

All these points are explained below.
The figures are informative, but minor corrections are needed, including improving Figure 1 arrows, standardizing data representation in Figure 3, adding the missing Y-axis value in Figure 5, and replacing the schematic graph in Figure 4 with a numerical table.

All these corrections in Figures 1, 3 and 5 have been made and Figure 4 was replaced with a table.

**Conclusions**

Reviewer #1: -The first paragraph of discussion should be mover to the end of introduction as it states justification and aim of the study. Please start the discussion by focusing on the main findings.

The first paragraph has been moved to the introduction and the discussion has been edited.

-Please rephrase the sentence "The centers of the lesions remained dry (see also Fig 2), and the more efficient immune processes involved in skin healing may have negatively affected the vitality of amastigotes at this site, preventing them from infecting the vector.” to make it clearer.

The sentence was rephrased to: The more efficient immune processes involved in skin healing in this natural host were reflected by the dry appearance of the lesion centres (see Figure 3), which may have negatively affected the vitality of amastigotes at this site, thereby preventing infection of the vector.

Reviewer #2: The conclusion would be much more impactful if articulated more clearly and directly.

We have edited the discussion to increase its clarity.

Reviewer #3: the study highlights the importance of using natural hosts to improve our understanding of parasite transmission dynamics, however the limitations regarding sample size and infection time points are not explicitly addressed and should be better discussed.

This text was added and linked to the second paragraph of Discussion: “Despite a low infectious dose, the vast majority (93 %) of *M. shawi* developed lesions that persisted for several weeks. As a result, the availability of material from asymptomatic ear pinnae was limited, comprising only two presymptomatic and one healed pinna, all of which exhibited a lower parasite load compared to symptomatic animals. However, the difference in parasite numbers was not statistically significant, which aligns with our previous study demonstrating that the presymptomatic *M. shawi* and animals with healed skin symptoms can still serve as infectious hosts for vector (10)”

The clarification to infection time points was included into Methods and Results, see below.

**Editorial and Data Presentation Modifications?**

Reviewer #3: Minor revisions

The text is concise and clearly describes the objectives and results of the project.

1- Introduction:

Line 16- The sentence should be revised: These reservoir animals are the main source of infection for vectors, phlebotomine sand flies (Diptera: Psychodidae), which act as vectors, transmitting parasites to humans, who are incidental hosts.

The sentence was changed to: “These reservoir animals are the main source of infection for phlebotomine sand flies (Diptera: Psychodidae), which act as vectors, transmitting parasites to other vertebrate hosts, including humans, who are incidental hosts.

Please expand the introduction. Specifically, include information on how P. papatasi serves as a specific vector for L. major, highlighting key aspects of factors influencing vector competence.

We have added this information to the introduction: “Phlebotomus papatasi is the principal vector of *L. major* over a wide geographical range that extends from North Africa to India, with breeding sites in rodent burrows (Maroli 2012). In this specific vector, parasite attachment to the midgut epithelium is controlled by the surface glycoconjugate lipophosphoglycan, which selectively binds to the midgut galectin receptor (Kamhawi 2006).”

2- Methods and Results

Experimental infections of rodents and Infectiousness of rodents at the microscale: (The experiment was terminated at 15–38 p.i. in Meriones shawi - )

It is unclear what infection time points were analyzed for M. shawi, and the reason for the long time gap between the selected post-infection intervals is not explained. Could you clarify the rationale behind these choices?

The text of Methods was expanded to clarify the choice of time intervals:

**Experimental infections of rodents:** “In *Meriones shawi,* the experiment was mostly terminated at week 30 p.i. (in 21 from 32 animals), but to cover a wider range of disease manifestations and to obtain samples from asymptomatic phases of infection, some animals were euthanized at different time intervals: from week 15 p.i., when lesions appeared, to week 38 p.i., when lesions were healed.”

**Infectiousness of rodents at the microscale**: “at weeks 15–16 p.i., when skin lesions started to appear and weeks 25–30 p.i., when lesions were fully developed).

The number of infected and asymptomatic animals analyzed appears to be low, with only three asymptomatic ears examined. Additionally, how does this sample size impact the robustness of the conclusions regarding parasite load dynamics in M. shawi?

Indeed, the number of three asymptomatic cases with proven infection that we have observed in gerbils is so small that it does not allow a relevant quantitative assessment (we are aware that for a fully relevant quantitative comparison, orders of magnitude more infected rodents would need to be studied, but this is technically and ethically impossible). However, the result that three asymptomatic individuals were able to demonstrate a relatively high parasite load is qualitatively significant, logically consistent with the interpretation put forward, and in our opinion worth reporting and discussing.

Fig 1 – The arrow in Figure 1 is not appearing. Please check and adjust the figure to ensure the arrow is clearly visible.
We apologize for the technical error; Figure 1 has been adjusted to show the arrows (now Figure 2)

In Figure 3, panel (E) appears to express the data as percentages, whereas panel (F) seems to use relative numbers. To ensure consistency and clarity, you should express all data in percentages.

Figure 3 has been adjusted so that both axes are in percentages (now Figure 4)

Specifically, 50.0% of the females that fed at the lesion centers and 58.3% of those that fed at the lesion margins acquired the infection (Fig 3F). The 50% infection rate is based on only four animals. Could the small number of insects used in this analysis influence the reliability of the results?
Although the results are formally described in terms of the number of cases and percentages, i.e., quantitatively, we are fully aware that the confidence intervals for the binomial distribution are very wide even in the case of sample sizes on the order of tens to hundreds of cases. Thus, our goal was simply to describe the actual results to provide the reader with qualitative insight. And that is what these data provide. The fact that the mice were able to transmit infection even when sand flies fed in the center of the lesion is worthy of mention. This occurred twice (in two mice) and is therefore unlikely to be a laboratory error.

Fig 4. Schematic of the trend of parasite loads in MBs collected from M. shawi pinnae during the experiment. It would be more valuable to present a table with the MB values found rather than a schematic graph.

The trend is now presented as Table 4.

Fig 5. Infection rates of P. papatasi females fed different infective doses. Please add the missing 10% value on the Y-axis

The Y-axis scale was adjusted.

**Summary and General Comments**

Reviewer #1: (No Response)

Reviewer #2: The authors highlighted the differences in infectiousness to sandflies between natural reservoir hosts and animal models. They found that using animal models to estimate the infectiousness of parasites to vectors tends to underestimate parasite transmission in leishmaniasis. They reported that only a few parasites from natural animal models could establish an infection in sandflies, in contrast to the much higher numbers observed in animal models needed to establish infection. These findings could enhance our understanding of Leishmania transmission.

Reviewer #3: In this study, the authors show an interesting difference in the distribution of Leishmania major amastigotes in the skin of Meriones shawi and BALB/c mice revealed differences in infectiousness to sand flies. These findings underscore the importance of natural host models for a better understanding of CL transmission and more accurate epidemiological modeling. So, the current study is on a topic of relevance and general interest to the readers of the journal presenting originality and importance of the contribution for the development of the field of study.

The authors of this article have extensive experience in Leishmania-sand fly interactions and have provided valuable insights into the transmission dynamics of Leishmania major, particularly the role of Meriones shawi as a reservoir host.
I think that a histological or microscopic examination of the three zones of ulcerative lesions in both BALB/c mice and M. shawi at the same time points could provide valuable insights into the spatial dynamics of parasite distribution and tissue response.

We thank the reviewer for the supportive evaluation of the manuscript, we appreciate it. Histological analysis would be a good complement to the study, but the gerbil ears were used for quantitative PCR analysis of parasite numbers and distribution, so there was no more material for histological/microscopical analysis. This would certainly be a good focus for further research, as would the details of the immunological response in the natural host. We believe that our paper could start more interest in this issue and initiate research in this direction.

While Meriones shawi is the natural reservoir host for Leishmania parasites, these animals remain in a laboratory setting for extended periods without exposure to sand flies and parasites. How do the authors relate the findings from this animal study to those in the wild, which experience higher exposure to sand-fly bites and parasite infections, concerning the parameters measured in the experiment? The immune response of hosts with frequent exposure might differ from those with no or limited exposure.

We thank the reviewer for this suggestion and the following text has been added to the discussion: “Rodents in nature are in constant contact with sand flies, which frequently nest in their burrows (6). Repeated exposure to multiple bites from uninfected sand flies affects the progression of infection. Vojtkova et al. (27) reported that *L. major*-infected BALB/c mice exposed to naïve sand flies at two-week intervals developed skin lesions more rapidly and exhibited a higher parasite load. In our experiment, animals were exposed to sand flies at five-week intervals during xenodiagnosis. Thus, under natural conditions, more frequent exposure to sand fly saliva may accelerate lesion development and increase parasite burden at affected sites. However, this hypothesis requires experimental validation, ideally using *M. shawi* or another natural host model”
