# Supplementary material for: Infectiousness of Leishmania major to Phlebotomus papatasi: differences between natural reservoir host Meriones shawi and laboratory model BALB/c mice
Source: PLoS Negl Trop Dis. 2025 Jun 16;19(6):e0013183. doi: 10.1371/journal.pntd.0013183 (PMC12201674; doi:10.1371/journal.pntd.0013183)
Supplement: Attachment — Submitted filename: Review report PLOS NTD 2025_2.docx [file pntd.0013183.s007.docx]

Re: Infectiousness of *Leishmania major* to *Phlebotomus papatasi*: differences between natural reservoir host *Meriones shawi* and laboratory model BALB/c mice

PNTD-D-25-00050

**Authors**: Vojtková et al.

**General comments**

The authors have addressed most comments, except for a few remarks.

**Comments**

“Rodents in nature are in constant contact with sand flies, which frequently nest in their burrows (6). Repeated exposure to multiple bites from uninfected sand flies affects the progression of infection. Vojtkova et al. (27) reported that L. major-infected BALB/c mice exposed to naïve sand flies at two-week intervals developed skin lesions more rapidly and exhibited a higher parasite load. In our experiment, animals were exposed to sand flies at five-week intervals during xenodiagnosis. Thus, under natural conditions, more frequent exposure to sand fly saliva may accelerate lesion development and increase parasite burden at affected sites. However, this hypothesis requires experimental validation, ideally using *M. shawi* or another natural host model”.

- May the authors consider how repeated exposure accelerates, rather than limits, the lesion's progression? May the authors justify why the immune system is not linked to repeated exposure and lesion progression?

Other comments

Under the introduction, the **first paragraph**

Cutaneous leishmaniasis (CL), characterized by a nonpainful…. **Nonpainful** needs curiosity by considering the patient's perspective. There is a pain.

Last paragraph of Introduction

To address this gap, we studied M. shawi originating from an endemic site in Algeria and BALB/c mice, a commonly used laboratory model for CL, both infected with a low dose of L. major. In previous work, we demonstrated that even asymptomatic individuals of M. shawi can serve as effective sources of infection for sand fly vectors (10). Here, we report the spatial distribution of parasites in infected ear pinnae at the microscale and the threshold number of L. major required for infection in the proven vector Phlebotomus papatasi.

- This statement remains unclear regarding why the authors don’t precisely state the study's objectives.

Table 2Table 2. Trend in parasite load between weeks 15 and 25 p.i. in MBs collected from the same M. shawi ear pinnae.

- No data to quantify instead of increased/stable?
